# Predicting Performances on Processing and Memorizing East Asian Faces from Brain Activities in Face-Selective Regions: A Neurocomputational Approach

**DOI:** 10.3389/fnhum.2020.00269

**Published:** 2020-07-29

**Authors:** Gary C.-W. Shyi, Peter K.-H. Cheng, S.-T. Tina Huang, C.-C. Lee, Felix F.-S. Tsai, Wan-Ting Hsieh, Becky Y.-C. Chen

**Affiliations:** ^1^Department of Psychology and Center for Research in Cognitive Sciences, National Chung Cheng University, Chiayi, Taiwan; ^2^Advanced Institute of Manufacturing with High-tech Innovations, National Chung Cheng University, Chiayi, Taiwan; ^3^Research Center for Education and Mind Sciences, National Tsing Hua University, Hsinchu, Taiwan; ^4^Department of Electrical Engineering, National Tsing Hua University, Hsinchu, Taiwan

**Keywords:** face processing, face memory, face selective brain regions, MVPA, SVM classifier

## Abstract

For more than two decades, a network of face-selective brain regions has been identified as the core system for face processing, including occipital face area (OFA), fusiform face area (FFA), and posterior region of superior temporal sulcus (pSTS). Moreover, recent studies have suggested that the ventral route of face processing and memory should end at the anterior temporal lobes (i.e., vATLs), which may play an important role bridging face perception and face memory. It is not entirely clear, however, the extent to which neural activities in these face-selective regions can effectively predict behavioral performance on tasks that are frequently used to investigate face processing and face memory test that requires recognition beyond variation in pose and lighting, especially when non-Caucasian East Asian faces are involved. To address these questions, we first identified during a functional scan the core face network by asking participants to perform a one-back task, while viewing either static images or dynamic videos. Dynamic localizers were effective in identifying regions of interest (ROIs) in the core face-processing system. We then correlated the brain activities of core ROIs with performances on face-processing tasks (component, configural, and composite) and face memory test (Taiwanese Face Memory Test, TFMT) and found evidence for limited predictability. We next adopted an multi-voxel pattern analysis (MVPA) approach to further explore the predictability of face-selective brain regions on TFMT performance and found evidence suggesting that a basic visual processing area such as calcarine and an area for structural face processing such as OFA may play an even greater role in memorizing faces. Implications regarding how differences in processing demands between behavioral and neuroimaging tasks and cultural specificity in face-processing and memory strategies among participants may have contributed to the findings reported here are discussed.

## Face Processing and Memory: Mechanisms Revealed by Behavioral Tasks

Face processing and memory entail not only encoding the perceptual input of a face upon its presence but also retrieving a relatively permanent representation despite variation in illumination, pose, or expression (Bruce and Young, 1986, 2012; Hole and Bourne, 2010).

### Aspects of Face Processing

Researchers generally agree that there are three aspects of face processing that eventually would lead to the creation of face representation (Maurer et al., 2002; Mondloch et al., 2002; for a review see Yang and Shyi, 2010). *Component* or *feature* processing refers to the way individuals process faces by encoding (nameable) facial features separately, such as eyes, nose, and mouth. *Configural* processing on the other hand refers to processing the spatial or metric information between facial features (e.g., the interocular distance, or distance between nose and mouth). Finally, *holistic* processing refers to the fact that people tend to process the face as an undifferentiated whole, simultaneously integrating all featural as well as inter-featural metric information that existed in a given face into a single perceptual representation (Tanaka and Farah, 1993, 2003; Rossion, 2008, 2009; McKone and Yovel, 2009; McKone, 2010).

There has been some controversy regarding the merit or usefulness of treating configural and holistic processing as distinct and completely separable face processes. While not attempting to resolve the controversy here, we are more inclined to take the view advocated by Rossion (2008, 2009, 2013), McKone (2010; see also McKone and Yovel, 2009), Tanaka and Gordon (2011, see also Tanaka and Farah, 1993, 2003), and Richler and Gauthier (2013, 2014), where holistic processing entails integrated processing of all features, nameable and non-nameable, as well as a variety of detailed metric relationships between and among them (McKone and Yovel, 2009), which results in a single face representation created by simultaneous integration and combination (see Kimchi, 1992, for an in-depth discussion on distinguishing between holistic and global properties in face or other hierarchically structured visual patterns).

Viewed this way, these researchers have also argued that the *composite* face task is perhaps the task most appropriate for assessing holistic processing. Composite face effect refers to the fact that occurs when recombining top and bottom halves of two faces would create the illusion of a face entirely different from either of its parent faces (Young et al., 1987; Hole, 1994; Rossion, 2013). When people are asked to judge the upper halves, which are identical, from two recombined faces, they are almost inevitably affected by the two different bottom halves and tend to perceive top halves as different (i.e., composite face illusion). This effect clearly demonstrates that people generally have a strong tendency to integrate upper and lower halves of a face into a single unit; in other words, people cannot perceive exclusively part of a face without being affected by other parts (Rossion, 2008, 2009, 2013). When two composite faces are (sufficiently) misaligned, however, the illusion disappears presumably because the integrated representation and continuation between the top and bottom halves of a face falls apart due to misalignment, which in turn allows participants to perceive either the top or bottom half of a face without being interfered by the other half (Tanaka and Farah, 1993; Farah et al., 1998) as a consequence of disrupting the law of good continuation (Richler and Gauthier, 2013; Rossion, 2013).

### Face Memory

In addition to the various aspects of processing that are involved in encoding a face upon its presentation, additional processing is needed to create robust and long-lasting representation such that a recurring face can be effectively retrieved and recognized. Early attempts in devising instruments for assessing the ability of face memory, such as Benton Facial Recognition Test (BFMT; Benton et al., 1983) and Recognition Memory Test for Faces (RMF; Warrington, 1984), have been criticized for providing non-face-related cues such that performance on these tests do not necessarily reflect the proficiency in face memory *per se* but rather more general memory ability. To remedy problems plaguing the existing tests, Duchaine and Nakayama (2006) developed and popularized the Cambridge Face Memory Test (CFMT), by getting rid of non-face information from test images and presenting them in various views and illuminations. These measures not only overcome shortcomings of the previous tests but also imitate the scenario of how people encounter faces on a daily basis, where variation in pose and illumination of any to-be-recognized face is the rule rather than exception. More specifically, there are three stages in CFMT where learned face images are tested with *identical* images, *novel* images where variations in viewpoint and/or illumination were added to the learned images, and novel images masked with visual *noise*. Judging from the overall response accuracy, difficulty systematically increases across the three stages, and participants have to truly learn and memorize target faces in order to correctly pick them out from distracters. Duchaine and Nakayama (2006) also tested prosopoagnosic patients and found those patients performed worse in the CFMT compared to normal participants, suggesting that CMFT could correctly identify those with deficits in face recognition and memory and hence constitutes a valid tool for assessing face memory ability.

### The Relationship Between Face Memory and Face Processing

Following the procedure of CFMT, Cheng Y. -H. et al. (2016) have recently created the Taiwanese version, called Taiwanese Face Memory Test (TFMT), using images from a Taiwanese face database (Shyi et al., 2013). In their study, Cheng Y. -H. et al. (2016) used TFMT in conjunction with the three aforementioned face-processing tasks to investigate age differences in face processing and memory. Like CFMT, TFMT was administered in three stages with increasing difficulty. The results of TFMT revealed a pattern almost identical to that found with CFMT (Duchaine and Nakayama, 2006). In addition, younger adults outperformed older adults in TFMT as well as in all three face-processing tasks. Furthermore, while younger adults exhibited reliable inversion effects in both component and configural tasks, older adults failed to do so except for the oldest subgroup (75 years and older) showing an inversion effect in the configural task. In contrast, older adults exhibited a pattern of interaction between alignment and congruency, similar to that found with younger adults for the composite task, demonstrating that both age groups can process faces holistically. Moreover, regression analyses on the relationship between face memory and face-processing performances for both age groups revealed that, while each face-processing task has its own share of holistic processing, they may be tapping into different aspects of holistic processing. Most important and somewhat surprising, holistic processing captured by the *component* task and its inversion effect, entailing subtle spatial relationship between local facial features and landmarks, may underlie face memory performance for both younger and older adults in Taiwan (Cheng Y. -H. et al., 2016). Taken together, besides showing distinct styles or strategies between younger and older adults in coping with the specific demands of each face-processing task, Cheng Y. -H. et al.’s (2016) findings suggest that both young and older participants appeared to rely on the same aspects of holistic and non-holistic processing revealed by the component task, for encoding and later retrieving memory for faces.

It should be pointed out, however, that while CFMT (and TFMT) embodies a form of face memory tailoring more closely to the perceptual aspect of acquiring and later retrieving the memory of a relatively novel face, it fails to address other forms of face memory that may involve recognizing faces with high familiarity such as those of celebrities (Shyi and He, 2011). Retrieving memory of highly familiar faces may well invoke other non-perceptual connections that were established in the process of familiarization. A case in point is a recent study by Moret-Tatay et al. (2020), who demonstrated that the associative links between face and name for celebrities can be asymmetrical, where the magnitude of priming from seeing a celebrity face to recognizing his or her name was greater than that from seeing a name to recognizing the corresponding face.

### Cultural Specificity and Differences in Face Processing

The ubiquity in the physical structure of a face, comprising a pair of eyes on the top, a nose in the middle, and a mouth at the bottom, situated within an ellipsoid, for people from different social and cultural background has allured researchers from early on to assume universal processing insofar as face identity is concerned (Yarbus, 1965; Bruce and Young, 1986, 2012). However, as pointed out by Henrich et al. (2010) critical review, many psychological effects, including fundamental aspects of cognitive and affective processing, differ for participants from different cultures. More specifically, Blais and his colleagues had reported evidence indicating that East Asian (EA) adults tend to preferentially fixate the central region (i.e., the nose) of a face, whereas Western Caucasian (WC) adults tend to fixate more on eyes and mouth regions during both learning and recognizing, and categorizing novel Eastern and Western faces (Kelly et al., 2010). Moreover, the consistent pattern of difference in eye movements was present regardless whether or not EA and WC participants were viewing faces from their or a different race (Blais et al., 2008).

The initial finding of cultural differences in face processing was later replicated and generalized by Kelly et al. (2010). Specifically, they compared EA (Chinese) participants and WC (British) participants and found essentially the same results reported by Blais et al. (2008). In addition, they also found consistent different patterns of eye movement between the Chinese and British participants in viewing other types of visually homogeneous objects such as sheep and greebles (Gauthier and Tarr, 1997). Most intriguingly, in a subsequent, cross-sectional study, Kelly et al. (2011) found a developmental trend where young children from China and UK demonstrated patterns of eye movement similar to those exhibited by the adults from their respective cultural background as they grew older. These findings clearly lend support to the notion that in a nontrivial manner, culture shapes face processing.

## Face Processing and Memory: Mechanisms and Brain Regions Revealed by fMRI Studies

Kanwisher et al. (1997) first identified the fusiform face area or FFA, which was located in the lateral part of the fusiform gyrus and specifically tuned to the processing of faces. Since then, numerous studies have been reported to uncover its properties, including the nature of representations it may contain. Aside from characterizing FFA as a specialized face module, it is important to point out that a broad consensus from many studies has emerged over the past two decades, arguing that face processing actually entails complex interactions among a network of brain regions (for recent reviews, see Duchaine and Yovel, 2015; Freiwald et al., 2016). Among them, three regions, namely, FFA (fusiform face area), OFA (occipital face area), and pSTS (posterior region of superior temporal sulcus), have consistently been identified and proposed to serve as the “core system” of face processing (Gobbini and Haxby, 2007; Haxby and Gobbini, 2011).

### Fusiform Face Area

Many studies have provided evidence for the notion that the main function of FFA is to structurally encode faces (for a review see Kanwisher and Barton, 2011). Liu et al. (2010), for example, showed that the FFA was sensitive to the basic physical structure of a face, including both the presence of face features (eyes, nose, and mouths) and proper configuration between them. Furthermore, using fMRI adaptation procedure, many studies have demonstrated that the FFA is sensitive to differences in face identity, and adaptation across repeated images of the same face is found even when those images differ in position (Grill-Spector et al., 1999), size (Grill-Spector et al., 1999; Andrews and Schluppeck, 2004), or spatial scale (Eger et al., 2004). In contrast, release from adaptation in the FFA was evident when participants indicated that they perceived a change in identity (Fox et al., 2009). These findings suggest that the FFA encodes structural aspects that are related to identity. It should be noted, however, that the FFA does not seem to represent face identity in a viewpoint-invariant manner. Benton et al. (2006), for example, found that the FFA exhibits substantial decrease in adaptation (i.e., release from adaptation) as the rotation angle between adaptation and test viewpoints increases. Ewbank and Andrews (2008), however, noted that while the release from adaptation was apparent in the FFA when unfamiliar, stranger faces were viewed at discrepant viewpoints, adaptation across viewpoint change was evident when viewing highly *familiar* faces such as celebrities.

### Occipital Face Area

The OFA, located upstream to the FFA and often assumed to be an earlier stage in the face network, sends its output to the FFA. As pointed out by Kanwisher and Barton (2011), studies have provided evidence consistent with such a view. For example, fMRI adaptation studies showed that the OFA is sensitive to changes in face stimuli regardless whether or not changes of face identities were perceived by the viewer, whereas the FFA is sensitive only to the perceived changes in identity (Fox et al., 2009). Moreover, Yovel and Kanwisher (2005) found that the magnitude of the OFA response was similar for upright and inverted faces, and there was no correlation across participants between the magnitude of the behavioral inversion effect and the difference in response of the OFA between viewing upright and inverted faces. In contrast, the FFA showed higher activity to upright than inverted faces, and this difference was correlated with the behavioral inversion effect. Finally, whereas the FFA responds to stimulus information about both face parts and configuration (Liu et al., 2010), the OFA is sensitive only to face parts (Liu et al., 2002).

### Superior Temporal Sulcus

According to Kanwisher and Barton (2011), while the FFA can be found in virtually all normal participants, face-selective regions in the superior temporal sulcus (fSTS) are less reliable, and are found in only half (Kanwisher et al., 1997) to three quarters among the participants (Fox et al., 2009). For this reason, fSTS has been studied less extensively than the FFA. Nonetheless, a number of studies have suggested important functional distinction between the fSTS and other face-selective regions in the cortex and have shown that the fSTS is involved in the processing of dynamic aspects of a face such as eye gaze, emotional expression, and speech. Using an fMRI adaptation paradigm, for example, Winston et al. (2004) tested for a neuroanatomical dissociation between identity and expression in face perception, and found that repeating identity across face pairs led to reduced fMRI signal in the FFA and *posterior* STS (pSTS), whereas repeating emotional expression across pairs led to signal reduction in a more *anterior* region of STS (aSTS) and not at all in the FFA. Their results provide neuroanatomical evidence for the distributed model of face processing and highlight a dissociation within the right STS between a caudal segment coding identity and a more rostral region coding emotional expression. In contrast, the adaptation study by Fox et al. (2009) also demonstrated that both the FFA and the pSTS showed release from adaptation when participants perceived a change in either identity or expression; however, the effect in pSTS occurred when participants were explicitly asked to attend to facial expression. Therefore, Fox et al. (2009) concluded that their results indicate functional overlap in the FFA and pSTS, with identity and expression signals in both regions, and they argued against a complete independence of identity and expression processing in regions of the core face-processing network.

It is worth noting that, in addition to the core system of the OFA, FFA, and fSTS, Collins et al. (2016) recently have pointed out an important role of the ventral anterior temporal lobe (vATL) in face processing. Specifically, they argued that an increasing number of studies have implicated the vATL in humans exhibiting both high-level perceptual and mnemonic functions. With respect to the perceptual function, the vATL may be the site at which view-invariant representation of a face is ultimately created and hence allows for identification of a face regardless of viewpoint variation. The mnemonic function of the vATL, on the other hand, entails a natural consequence of the high-level perceptual function in that the identity-invariant representation can be used to match representation from long-term memory for recognition of a familiar face and access to semantic information associated with the familiar face (Bruce and Young, 1986, 2012).

## The Present Study

In the present study, we aimed to examine the extent to which brain activities in the face-selective regions can predict performances in behavioral tasks that have been proposed and used to reveal mechanisms underlying face processing and face memory, especially when non-Caucasian EA (Taiwanese) faces are involved. Specifically, we investigated how brain activities in the face-selective areas such as the FFA, OFA, pSTS/fSTS, and vATL of an observer can be used to predict his or her performance on tasks tapping component processing, configural processing, holistic processing, as well as face memory. To that end, we identified during a functional scan the core face network by asking participants to perform a one-back task, while viewing either static images or dynamic videos. Besides, they were asked to perform a variety of face-processing tasks outside the scanner, including the TFMT, component task, configural task, and composite task (Cheng Y. -H. et al., 2016). In addition to the univariate GLM approach to analyzing neuroimaging data, we also adopted the multi-voxel pattern analysis (MVPA) approach (Norman et al., 2006) to further elucidate whether and how brain activations in the traditional face-selective areas as well as other various areas can jointly be used to predict participants’ performance on face processing and face memory.

## Method

### Participants

Forty-four college students (24 females and 20 males, ranging from 19 to 35 years old, with a mean age of 21.98 ± 2.65 years) from the National Chung Cheng University in Chiayi County and the National Cheng Kung University in Tainan City of Taiwan were recruited to participate in the study. All participants had normal or corrected-to normal vision and were recruited, and all were native Chinese Mandarin speakers with no history of psychiatric or neurological disorders. Informed consent approved by the Human Research Ethics Committee of the National Cheng Kung University was obtained from all participants prior to their participation. Twenty-seven participants underwent MR scanning first and were then called back to perform the behavioral tasks, and vice versa, for the remaining 17. The delay between the MR scan and behavioral tasks varied from 7 to 76 days.

### Behavioral Tasks

Four tasks were performed outside the scanner for the behavioral part of the study. Of them, three tasks, namely, component, configural, and composite, were to assess performance on various aspects of face processing, and one test, the TFMT, was to assess performance on face memory. We described each task in detail below.

#### Face-Processing Tasks

The face images used in the three face-processing tasks were drawn from the Taiwanese face database by Shyi et al. (2013). Twelve individuals (six males and six females) were chosen from the database, and four of them, comprising two males and two females, were used as target faces for each of the three tasks. Posed with neutral expressions, all faces were cropped so that no hair was visible and facial blemishes were removed. The size of face images used in the component and configural tasks were 14.5 cm in length and 11 cm in width, extending a visual angle of approximately 13.8° × 10.5° at a viewing distance of approximately 60 cm, and those used in composite task were 16.2 cm in length and 12.4 cm in width, resulting in a visual angle of about 15.5° × 11.8°.

Each trial of the three face-processing tasks entailed presentation of a pair of faces on the two sides of a display screen. The faces were presented with a slight vertical spatial displacement. For component and configural tasks, the horizontal distance between two face images was 80 pixels (4.4 cm), and the vertical distance between the two faces was 91 pixels (5.1 cm); for the composite task, the horizontal distance between two face images was 60 pixels (3.2 cm), and the vertical distance between two faces was 91 pixels (5.1 cm). Upon seeing the face images, participants were told to judge whether they were the same or different in accordance with the response criterion for each task. For both the component and configural tasks, participants judged whether the two faces were exactly identical; for the composite task, they were to judge whether or not the top parts of displayed faces were exactly the same.

**Figure 1:**
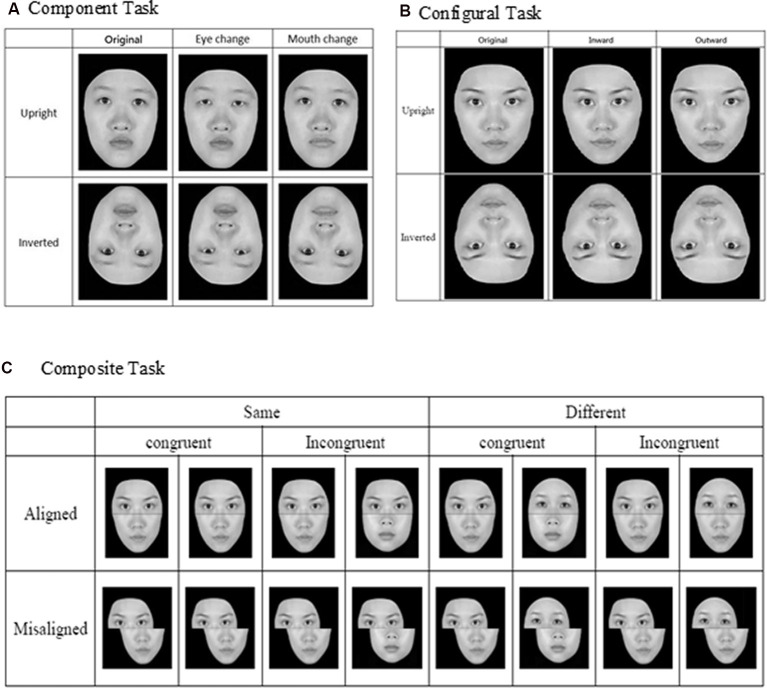
Face stimuli and design for the three face-processing tasks: **(A)** component task, **(B)** configural task, and **(C)** composite task (adapted from Cheng Y. -H. et al., 2016, with permission).

For the component task, the pair of faces could be identical to or different from each other, and when they were not identical, the faces differed either in terms of eyes or mouths (see Figure 1A). Three within-participant variables were manipulated for the component task, including orientation (upright or inverted), changed component (eyes or mouth), and identity (same or different). For the configural task, the face images were again either identical to or different from each other, and when they were different, they differed in terms of the configuration (i.e., distances) between the two eyes and between the nose and mouth. Specifically, as illustrated in Figure 1B, the distances between the two eyes and between the nose and mouth were either contracted or expanded by 8% in comparison to the original intact images. As for the component task, faces for the configural task were presented either upright or inverted. Note that the design and manipulation in both the component and configural tasks allowed us to measure participants’ performance in terms of hit rate (responded “same” when the faces were identical) and false alarm rate (responded “same” when the faces were different), and we applied the signal-detection theory to convert those measures into *d′* for further analyses.

Finally, for the composite task, we adopted the complete design proposed by Gauthier and Bukach (2007; see Figure 1C) to control for response bias associated with the partial design. Participants’ responses from the various conditions also can be converted into *d′* for further analysis. As in the previous studies that have employed the complete design, we expected to find congruency effect (i.e., the difference in *d*′ between congruent and incongruent trials) in the aligned condition to be greater than that in the incongruent condition (i.e., an interaction between congruency and alignment; Richler et al., 2011; Ross et al., 2014).

In each trial of the three face-processing tasks, a fixation point (“+”) of 0.43° × 0.28° was first presented for 500 ms, followed by the simultaneous presentation of two faces on upper left and lower right side (or upper right and lower left) of the display screen. Participants were asked to press either the [Z] key or [/] key on a standard keyboard to indicate whether those two faces were the same or different, and the key assignment was counterbalanced across participants. The face images remained on the screen until the participants responded. After an inter-trial interval of 500 ms, the fixation reappeared, signaling the start of the next trial. For both the component and configural tasks, there were altogether 96 trials, representing a combination of target faces, orientation, identity, location, and alteration. For the composite task, there were altogether 128 trials, representing a combination of target faces, congruency, identity, alignment, and location.

#### Taiwanese Face Memory Test

**Figure 2:**
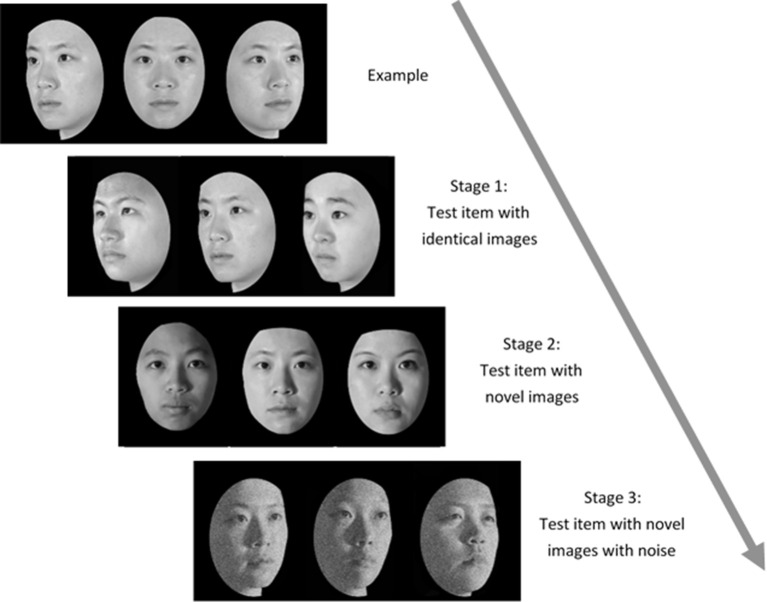
The three-staged procedure of the Taiwanese Face Memory Test (TFMT; adapted from Cheng Y. -H. et al., 2016, with permission; see text for details).

Administration of the TFMT (Cheng Y. -H. et al., 2016) was separated into three stages (see Figure 2). In Stage 1, participants studied three images from the same model in the frontal, left 1/3 profile, and right 1/3 profile views, respectively, and each was sequentially presented for 3 s. After studying the three images, three test images were presented and participants were asked to pick out the individual whom they just saw. Each test trial consisted of three face images of the same view and illumination, one of which was identical to the studied images. Two more test items followed, and each consisted of one of the studied faces along with two distractor faces. In Stage 1, participants learned 18 different images of six models (three males and three females) and tested for 18 times. In Stage 2, participants were first presented with a review comprising the frontal images of the six targets for 20 s. Following the review, participants were presented with 30 three-alternative forced-choice test items (6 target faces × 5 presentations). All were novel images in which the lighting, pose, or both varied. In Stage 3, participants were presented with the review images again for 20 s. Following that, 24 test items, each comprising three face images, were presented. These items consisted of novel images, and four levels of Gaussian noise (15, 30, 45, and 60%, respectively; Cheng Y. -H. et al., 2016) were added to the face images (6 target faces × 4 levels). All the face stimuli of TFMT were drawn from the database created by Shyi et al. (2013).

### Neuroimaging Tasks

#### Stimulus Materials

**Figure 3:**
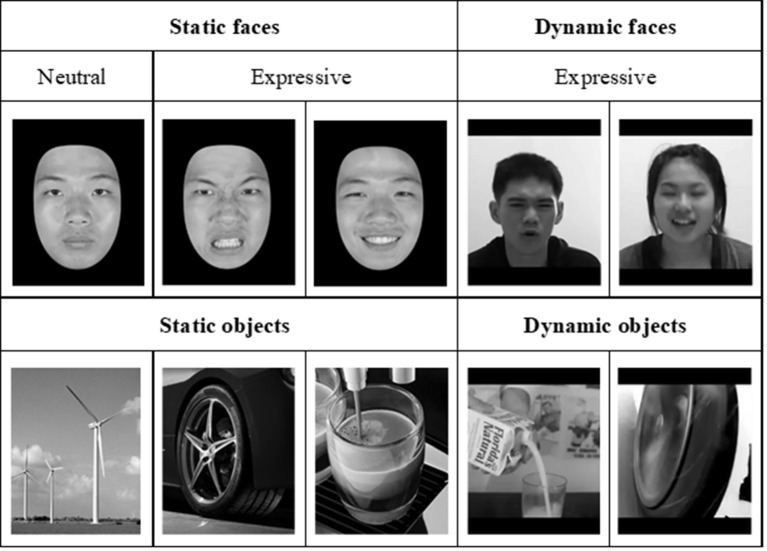
Examples of neutral and expressive face stimuli, which were selected from the face database by Shyi et al. (2013) and dynamic face videos selected from Huang et al. (2014). The static object images and dynamic object videos, similar to those used by Fox et al. (2009), were collected over the internet.

For the neuroimaging part of the study, we employed two sets of stimuli, the *dynamic* stimuli for localizing face-selective regions of interest (ROIs) and *static* stimuli for examining: (a) how face-selective regions would process face stimuli in contrast to non-face stimuli; and (b) whether and how those differences in brain activities can be used to predict performance on the behavioral tasks of face processing and face memory. As illustrated in Figure 3, the static neutral and expressive static face images were selected from Shyi et al. (2013), and the dynamic videos portraying dynamic facial expressions were selected from Huang et al. (2014). Each video depicted a silent rendition of uttering an emotional expressive sentence, and there were two kinds of face images, the neutral and expressive, both of which were selected from Shyi et al. (2013). The images of objects were gathered from the internet.

#### Experimental Design and Procedure

**Figure 4:**
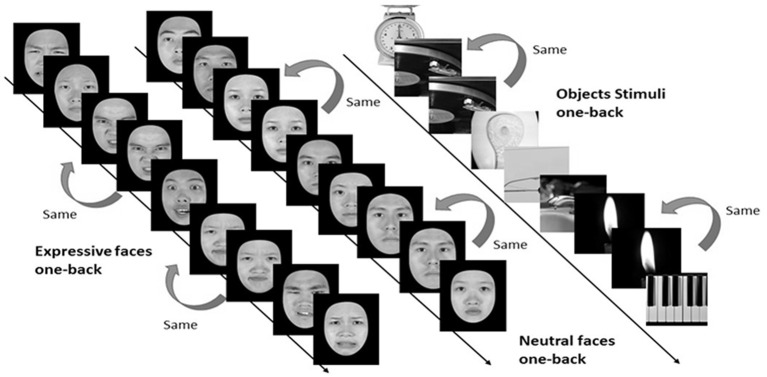
Three categories of static stimuli, namely neutral faces (the left stream), expressive (the middle stream) faces, and objects (right stream), were used for the one-back working memory task in the present study (see text for details).

In the *one-back* working memory (WM) task, participants viewed static images of faces and objects in separate blocks, and were to press a response button whenever the currently displayed image was identical to the immediately preceding one (see Figure 4). For the one-back WM task, 18 image blocks and 17 fixation blocks were alternated. The task began and ended with a fixation block, where only a fixation point was presented. Each block lasted for 12 s. Six blocks of stimuli from the three categories, namely, objects, neutral faces, and expressive faces, were presented in a counterbalanced order, resulting in a total of 18 blocks. Each block consisted of 15 images, 12 novel and three repeated ones. Facial images were cropped to conform to a frame of 400 × 400 pixels and presented on the screen for 500 ms with an inter-stimulus interval (ISI) of 300 ms. It took 444 s for participants to complete the one-back WM task.

In order to effectively localize the face-selective brain regions of FFA, OFA, STS, and vATL, participants were asked to undertake the dynamic face localizer task (Fox et al., 2009) after completing the one-back WM task, where static face and object stimuli were used. During dynamic localizers, participants viewed short videos comprising objects and faces in separate blocks. The face videos (each video portrayed an emotional facial expression, including angry, disgusted, fearful, happy, neutral, sad, and surprised) unfolded over time without sound tracks. The videos of objects were similar to those used by Fox et al. (2009) and were collected from the internet. For dynamic localizers, participants also performed a one-back task. Sixteen video blocks and 15 fixation blocks were alternated. Eight video blocks of faces and objects were presented in a counterbalanced order, 16 in total. Each video block consisted of six videos, five novel and one repeated. All videos were presented centrally for 1,800 ms with an ISI of 200 ms. Like the stationary one-back WM task, the dynamic localizer also began and ended with a fixation block. Dynamic stimuli of each block were resized to a frame of about 400 × 400 pixels. It took a total of 396 s for participants to complete the dynamic localizer tasks.

### Image Acquisition

A General Electric 3T scanner with an 8-channel phase-array head coil at the Mind Research and Imaging Center of National Cheng-Kung University was used to acquire brain images. Functional images were acquired in the form of T2*-weighted transverse echo planar images (EPI) comprising 40 axial slices, with a repetition time (TR) of 2 s, an echo time (TE) of 33 ms, a FOV of 192 × 192 mm^2^, an in-plane resolution of 3 × 3 mm, a slice thickness of 3 mm, and a voxel size of 3 × 3 × 3 mm^3^. Slices were oriented parallel to each participant’s anterior and posterior commissure (AC–PC) line, covering the whole brain. In addition, a high-resolution T1-weighted 3D-SPGR anatomical scan was acquired for the purpose of co-registration between structural and functional images and for anatomically localizing brain regions for functional activations.

### Data Analyses

Functional images were pre-processed and analyzed using SPM8 (Wellcome Department of Cognitive Neurology, London, UK). Pre-processing entailed slice timing correction (only for the recognition stage of fMRI images), spatial realignment, co-registration of binding EPI and T1 images, normalization to the Montreal Neurological Institute (MNI) template, and image smoothing with 6-mm full width-half maximum (FWHM) Gaussian kernel.

**Figure 5:**
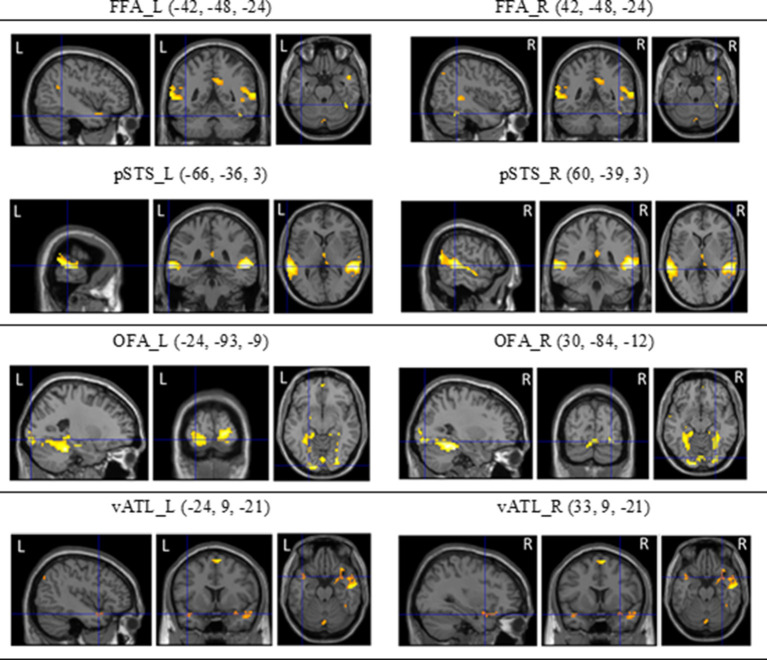
The regions of interest (ROls) of the core system for face processing [i.e., occipital face area (OFA), fusiform face area (FFA), superior temporal sulcus (STS), and ventral anterior temporal lobe (vATL)] on both hemispheres and their coordinates (see Fox et al., 2009; Haxby et al., 2014) derived from dynamic face localizers.

To localize the face-selective brain regions, we first analyzed fMRI data from dynamic localizers. Using the subtraction analysis within SPM8, we assessed average activations across participants by carrying out a two-step analysis. For the first-level individual analysis, each participant’s data were analyzed with a fixed-effect model to create contrasts between conditions of interest. For the second-level group analysis, we carried out one-sampled *t* tests on the results acquired from the contrasts of fixed-effect analysis of individual participants. Statistical maps of dynamic face localizers for group analyses were thresholded at *p* < 0.05 (FDR corrected). A mask of 5-voxel radius was applied to all statistical maps. The SPM yielded results in the stereotactic space of the MNI brain template, as shown in Figure 5.

For region-of-interest (ROI) analyses, we defined ROIs based on the brain regions from the dynamic face localizers, and from these ROI areas, we extracted BOLD percent signal change from the one-back processing task using the MarsBar procedure (Brett et al., 2002)^1^. The percent signal changes in the expressive faces, neutral faces, and objects were calculated, as were correlations between the difference in the activation of ROIs and behavioral performances on the three face-processing tasks and the TFMT. A *p*-value of less than 0.05 was considered significant.

## Results and Discussion

**Figure 6:**
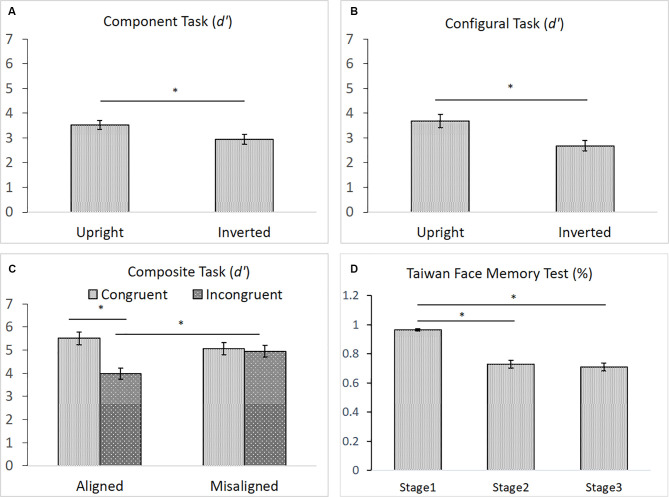
**(A)** The mean *d′* (and standard error) for the component processing task, **(B)** the mean *d′* (and standard error) for the configural processing task, **(C)** the mean *d′* (and standard error) for the composite processing task, and **(D)** the mean accuracy (and standard error) of the three stages on TFMT (*N* = 44; *p < 0.05; see text for details).

As shown in Figures 6A–D, for the component task, the difference in performance between upright (*M* = 3.53) and inverted (*M* = 2.95) faces was significance, *F*_(1,43)_ = 6.911, $\eta_{\text{p}}^{2}$ = 0.140, *p* < 0.05, indicating the presence of an inversion effect in the component task (Figure 6A). Likewise, for the configural task, the difference in participants’ performances between upright (*M* = 3.68) and inverted (*M* = 2.68) faces task also reached significance, *t*_(43)_ = 4.396, *p* < 0.001, indicating the presence of the inversion effect in the configural task (Figure 6B). For the composite task, both the main effect of congruency and its interaction with alignment were significant, *F*_(1,43)_ = 14.334, $\eta_{\text{p}}^{2}$ = 0.250, *p* < 0.001, and *F*_(1,43)_ = 8.095, $\eta_{\text{p}}^{2}$ = 0.158, *p* < 0.01, respectively. However, the main effect of alignment was not, *F*_(1,43)_ = 2.076, $\eta_{\text{p}}^{2}$ = 0.046, *p* = 0.157. Participants performed significantly better with congruent (*M* = 5.51) than with incongruent (*M* = 3.97) trials in the aligned condition, *t*_(43)_ = 5.314, *p* < 0.001, but they performed equally well with the congruent (*M* = 5.07) and incongruent (*M* = 4.95) trials in the misaligned condition, *t* < 1 (Figure 6C). Finally, similar to those reported by Cheng Y. -H. et al. (2016), participants’ mean accuracy of identifying the same face images in Stage 1 (*M* = 0.97) on the TFMT was much higher than that for recognizing faces for both Stages 2 (*M* = 0.73) and 3 (*M* = 0.71), *t*_(43)_ = 8.56, *p* < 0.001, *t*_(43)_ = 9.34, *p* < 0.001, and *t*_(43)_ = 9.77, *p* < 0.001, respectively (Figure 6D). Furthermore, like Cheng Y. -H. et al. (2016), there was no significant difference between performances on Stages 2 and 3, *t*_(43)_ = 1.11, *p* > 0.27.

**Figure 7:**
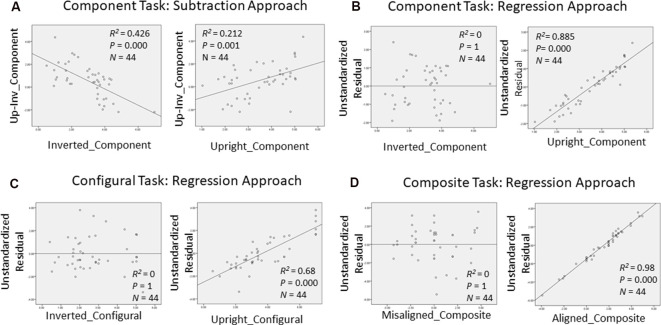
Measurements of holistic processing for the three face-processing tasks. For both the component task and configural task, inverted trials served as the control condition, while upright trials served as the condition of interest; for composite task, misaligned trials served as the control condition, while the aligned trials served as the condition of interest. **(A)** Using subtraction to measure the holistic processing for the component task; **(B)** using regression to measure the holistic processing for the component task; **(C)** using regression to measure the holistic processing for the configural task; and **(D)** using regression to measure the holistic processing for the composite task.

In order to gain further insight as to how face recognition performance on TFMT may be accounted for by those on face-processing tasks, we decided to adopt the regression approach to individual differences suggested by DeGutis et al. (2013) in their recent study. Following DeGutis et al. (2013), we first applied both subtraction and regression analyses to the component task, where we treated upright trials as the condition of interest comprising mostly elements of holistic processing (HP), and inverted trials as the control condition, which presumably reflect non-HP measurements. With the subtraction approach, as illustrated in Figure 7A, we found that the difference scores correlated negatively with the inverted trials (*p* < 0.001), and positively with the upright trials (*p* < 0.05). In contrast, with the regression approach (Figure 7B), residuals correlated strongly with the upright trials (*p* < 0.0001), but did not at all correlate with the inverted trials (*p* = 1), indicating that residuals may represent a purer estimate of HP element in the component task (Cheng Y. -H. et al., 2016). We applied the same regression approach to the configural task (Figure 7C) and composite task (Figure 7D) as well, and found results replicating those reported by Cheng Y. -H. et al. (2016).

We then examined the relationship between the three face-processing tasks and the TFMT to see the extent to which performance on the latter can be predicted by those on the former (see DeGutis et al., 2013; Cheng Y. -H. et al., 2016). As shown in Table 1, only the HP measurement derived from the regression approach (i.e., unstandardized residuals) for the component tasks correlated significantly with Stage 2 performances on TFMT, whereas residue-based HP measurements for neither the configural task nor the composite task correlated significantly with TFMT, *p*s > 0.05.

**Table 1:** The correlations between performances on the three stages (1, 2, and 3) of the TFMT and holistic processing measures of three face-processing tasks derived regression residues (*N* = 44).

	TFMT		
Task (residual)	Stage 1	Stage 2	Stage 3
Component	0.132	0.472**	0.289
Configural	−0.017	0.148	0.050
Composite	−0.032	0.051	0.123

**p < 0.01.

Finally, we examined the relationship between the BOLD signal change of ROIs and behavioral performances on the three face-processing tasks and TFMT. As shown in the Table 2, results revealed that participants with greater BOLD signals in the right FFA (positively correlated) and the left ATL (negatively correlated) demonstrated better performance on the holistic processing on component processing task, but not on the configural task or on the composite task. By correlating the face recognition ability from the three stages of TFMT with the participants’ BOLD signal change under a different condition during one-back processing tasks, we found that only the right ATL (expressive face > neutral face) was significantly negatively correlated with the performances on Stages 2 and 3 of TFMT.

**Table 2:** The correlations between BOLD signal change of face-selective ROIs as a function of contrasts between expressive faces, neutral faces, and objects and behavioral performance on the face-processing tasks and face-memory test (TFMT).

	rFFA (*E > O*)	lFFA (*E > O*)	rFFA (*E > F*)	lFFA (*E > F*)	rpSTS (*E > F*)	lpSTS (*E > F*)	rvATL (*E > F*)	lvATL (*E > F*)
Component*_Residual_*	−0.038	0.134	0.192	−0.041	0.019	0.031	−0.064	−0.287
Configural*_Residual_*	0.012	0.277	0.124	0.099	0.100	0.074	0.136	0.025
Composite*_Residual_*	−0.297	−0.020	−0.023	0.072	0.041	−0.073	−0.338*	−0.109
TFMT*_Stage1_*	−0.144	−0.097	0.080	0.017	0.055	0.119	−0.063	0.113
TFMT*_Stage2_*	−0.030	0.012	−0.099	−0.116	−0.187	−0.058	−0.229	−0.390**
TFMT*_Stage3_*	−0.085	0.008	−0.007	−0.054	−0.144	−0.109	−0.182	−0.276

Note: FFA, fusiform face area; pSTSE, posterior superior temporal sulcus; vATL, ventral anterior temporal lobe; *E > O*, expressive faces minus objects; *F* > *O*, neutral face minus objects; *E* > *F*, expressive faces minus neutral faces; *p < 0.05; **p < 0.01.

## Interim Summary

In the present study, we largely replicated the findings recently reported by Cheng Y. -H. et al. (2016) regarding the behavioral tasks on face processing and face memory insofar as young participants are concerned. For example, an inversion effect was found for both the component and configural tasks, so was the interaction between congruency and alignment, which strongly implicates holistic processing when the top and bottom parts of a face were aligned than when they were misaligned. The results on TMFT also closely replicated those reported by Cheng Y. -H. et al. (2016): young participants performed almost flawlessly in Stage 1, where performance largely depends on the encoding and remembering specific face images. The performance in Stage 2 was about 20% inferior to that in Stage 1, indicating the challenge and difficulty when newly acquainted faces undertake alterations in illumination, pose, or both. The addition of visual noise in Stage 3, however, failed to further exacerbate the performance observed in Stage 2, implicating that the TFMT may be shortened in the future to include only the first two stages to achieve more efficient assessment of face memory (Cheng Y. -H. et al., 2016).

On the other hand, the brain activities at the core-system face-processing areas, including OFA, FFA, pSTS/fSTS, and vATL in both hemispheres, appeared to be somewhat limited in their capacity of predicting behavioral performances. A number of reasons may be able to explain why that was the case: First, the fact that we used one-back WM task with dynamic stimuli for localizing the face-selective ROIs and static one-back task for assessing brain activities of those ROIs when engaged in face processing may have inadvertently reduced the likelihood of predicting behavioral performances from brain activities. Specifically, the three face-processing tasks may place minimal demand on working memory because in each trial of the three tasks, a pair of faces were displayed simultaneously such that the processing under scrutiny has little to do with memory. On the other hand, the face memory test—TFMT—does require working and perhaps even long-term memory in order to perform the test adequately. Therefore, it may not be too surprising that the brain activities from the ROIs showed their predictability primarily in performance on the TFMT (Stage 2 in particular, see Table 2). Another possibility is that brain activities of more than the core-system regions need to be taken into account in order to provide an adequate prediction of the behavioral performance. That is, there are aspects of face processing and memory involved in the behavioral tasks that were not completely captured by the brain activities of the core-system areas. To evaluate the latter possibility, we performed multi-voxel pattern analysis (MVPA; Norman et al., 2006) on brain activities recorded from a total of 19 ROIs, including the eight ROIs from the core-system of face processing that were examined so far and another 11 ROIs from what Haxby and colleagues have dubbed the extended system related to face processing (Haxby et al., 2000; Haxby and Gobbini, 2011). Of note, we only included the behavioral results from the TFMT to see whether and how brain activities of 19 ROIs may provide better predictions on face memory.

## MVPA of Brain Activities in Predicting Performances on Face Memory

**Figure 8:**
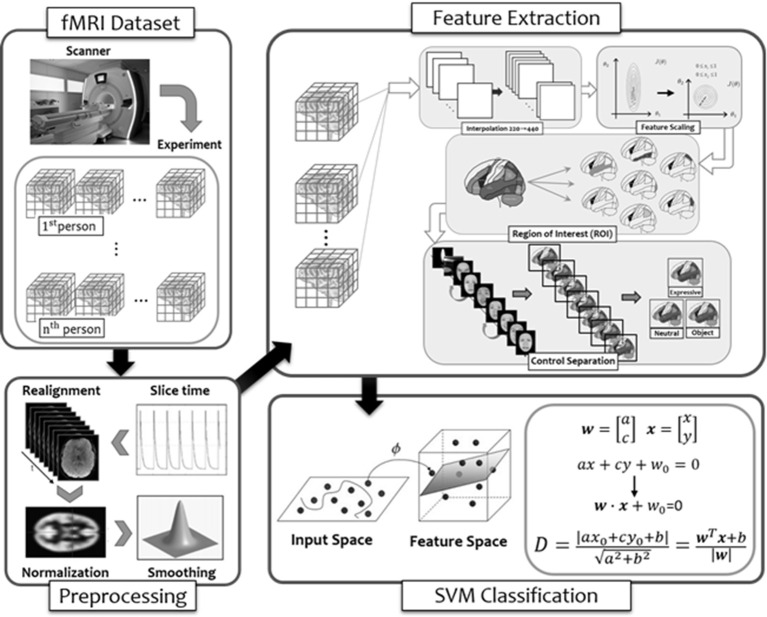
The flow diagram illustrated the steps of multi-voxel pattern analysis (MVPA) in the present study (see details in text).

Our attempt at MVPA in order to see whether activities in a broader selection of brain regions identified in the larger literature to be face-selective (Haxby and Gobbini, 2007; Duchaine and Yovel, 2015; Freiwald et al., 2016) can provide better prediction on performance in face memory test (i.e., TFMT) is summarized with the flow diagram in Figure 8, and we detailed each step below.

## Method

### fMRI Signal Preprocessing and ROI Selection

As noted earlier, MRI scanning (structural and functional) was conducted on a 3T scanner at the NCKU, and the functional scanning *via* EPI captured one image every 2 s (i.e., TR = 2 s). To consecutively represent data from entire brain, we performed temporal interpolation to generate an image sample at 1-s time step to handle the varying time length of visual frame within each stimulus. Interpolation was performed on the fMRI raw time series both to determine the activation value of voxels intermediate to the raw scanning data during optimization of the spatial transformation and to produce the final scanning session data once an accurate spatial transformation correcting for the participant was determined.

One concern in analyzing the raw fMRI data was that the level of signals may be substantially different between voxels due to the physiological properties of fMRI scanning. This constitutes a problem when comparing effect size of voxels and is more serious for analyzing data from multiple participants because the signal strength may vary substantially at the corresponding voxels. To achieve better performance and comparison across voxels, we transformed the fMRI time-series signals by applying the baseline Z normalization, where the BOLD signal of each voxel for each time slice was mean-centered by subtracting the mean signal strength and divided by standard deviation to reduce signal variations.

In order to select the ROIs encompassing both the core and extended system of face processing (Haxby and Gobbini, 2011), we first applied the AAL (Anatomical Automatic Labeling; Tzourio-Mazoyer et al., 2002) masks to split the entire brain into 90 regions. We then selected 19 regions, listed in Table 3, that have been identified by the past literature as playing some roles in the various aspects of face processing and face memory. The ROIs and their coordinates were based on two previous studies from our lab using the same dynamic localizers as in the present study for locating brain areas exhibiting face selectivity regions (Cheng P. K.-H. et al., 2016; Chen et al., 2017). Using the MarsBar tool for SPM^1^, we built each ROI by first defining the centroid of the sphere with 19 coordinates. With 5 mm as the sphere radius, the total volume for each ROI was approximately 648 mm^3^, and each ROI contained a cluster size of about 81 voxels (2 × 2 × 2 mm^3^/per voxel) associated with maximum face selectivity. Table 3 shows the list and numbering of the 19 ROIs thus selected and used for MVPA in the present study.

**Table 3:** The list of 19 ROIs that were selected based on past literature and the voxel activations that were used for computation analyses (IFG/OFC, inferior frontal gyrus/orbitofrontal cortex; ATL, anterior temporal lobe; FFA, fusiform face area; mSTS, medial superior temporal sulcus; OFA, occipital face area; OP junction, occipital-parietal junction; pSTS, posterior superior temporal sulcus).

Number	Hemisphere	Label(s)	*X*	*Y*	*Z*	ROI source
01	Right	IFG/OFC	36	24	-21	Cheng P. K.-H. et al. (2016)
02	Left	Amygdala	-21	-6	-15	Cheng P. K.-H. et al. (2016)
03	Right	Amygdala	21	-6	-15	Cheng P. K.-H. et al. (2016)
04	Left	ATL Pole	-42	9	-21	Cheng P. K.-H. et al. (2016)
05	Right	ATL Pole	33	9	-22	Cheng P. K.-H. et al. (2016)
06	Left	Calcarine	-10	-90	10	Chen et al. (2017)
07	Right	Calcarine	10	-84	8	Chen et al. (2017)
08	Left	FFA	-42	-48	-24	Cheng P. K.-H. et al. (2016)
09	Right	FFA	42	-48	-24	Cheng P. K.-H. et al. (2016)
10	Left	Hippocampus	-20	-30	-8	Chen et al. (2017)
11	Right	Hippocampus	20	-30	-4	Chen et al. (2017)
12	Left	mSTS	-60	-24	-6	Cheng P. K.-H. et al. (2016)
13	Right	mSTS	60	-21	-6	Cheng P. K.-H. et al. (2016)
14	Left	OFA	-24	-93	-9	Cheng P. K.-H. et al. (2016)
15	Right	OFA	30	-84	-12	Cheng P. K.-H. et al. (2016)
16	Right	OP Junction	46	-68	2	Chen et al. (2017)
17	Right	Precuneus	3	-69	33	Cheng P. K.-H. et al. (2016)
18	Left	pSTS	-66	-36	3	Cheng P. K.-H. et al. (2016)
19	Right	pSTS	60	-39	3	Cheng P. K.-H. et al. (2016)

*Note: The numerical order was arbitrarily assigned to the selected ROIs primarily for the purpose of label ID*.

### Feature Extraction and Representation

The goal of the MVPA was to examine the extent to which the brain activities of the 19 ROIs, while participants performed the tasks involving static faces (neutral and expressive) and static objects, can be used to predict face memory performance on the TFMT, where individual participants’ performances were binary labeled into the above- or below-mean group. To that end, we first extracted the voxels recorded under each stimulus type to construct the feature-level representation. Because the neuroimaging runs of different stimulus types entailed different lengths of duration and resulted in a varying number of time series outputted from MRI scanning, we carried out *max* and *mean* pooling over each of three stimulus types as participants’ feature representation under a specific task. The result showed that the maximum function represented the most activated voxels, whereas the mean function represented the average activation response of voxels. We then concatenated the result of voxels with maximum activation to be the final fMRI feature representation for each participant.

#### Forward and Backward Selection (F&B)

Two popular data-driven approaches to model building are the greedy algorithms of F&B to arrive at the feasible feature representation, where evaluation of the predictability of a subset of variables is carried out by either adding or deleting one variable at a time. In practice, the forward selection begins with an empty selection of attributes, and in each round, it would add an unused attribute and test for performance. That is, select the first variable that yields the highest performance (e.g., face recognition rate among all attributes). Then, select the attribute among all unselected attributes one at a time, and together, the selected attributes presumably would lead to the highest performance. The process is repeated until it has selected enough numbers of attributes, or until the resulted performance is adequate. Unlike forward selection, the backward elimination begins with the full set of attributes, and iteratively removes the least useful attribute, one at each time, until the highest or good enough performance is achieved.

#### Feature Selection (FS)

In order to figure out which ROIs contributed more (or less) to the prediction of behavioral performance in the process of forward selection and backward selection mentioned above, the FS method based on ANOVA *F*-value between features and labels for classification was applied for each loop in the forward selection and backward selection (Dash and Liu, 1997). In our implementation, we consider a range of percentage of including different numbers of features for purpose of training our model, in an attempt to achieve the local optimal result in each loop. We backtracked the brain regions with features that yielded the local maximum results and counted the number of times each region was selected. Furthermore, the counts were normalized by the original feature size of each region to eliminate the effect from uneven regional data. Subsequently, the count result in each loop using forward and backward method was recorded.

### SVM Classification

As in many other MVPA studies in the literature, the selected classifier for training and recognition was a linear-kernel support vector machine (SVM) with the penalty parameter of *C* = 1. We compared the classification accuracies between feature representations based on three sets of ROIs as input for training, namely, the set of core-systems ROIs (i.e., the baseline, BL), the ROIs based on F&B, and those based on FS.

## Results and Discussion

In what follows, we first described the results of feature extraction, in particular the ROIs chosen based on forward and backward (F&B) selection as well as FS. We then reported the results of prediction accuracy based on the binary SVM classification on the TFMT (i.e., above or below the overall mean performance), where prediction accuracy was defined as an unweighted average of hit (correct positive response) and correct rejection on classifying TFMT performance, with the evaluation scheme of leaving-one-participant-out cross-validation. In particular, we highlighted how the prediction accuracy using SVM varied between the three sets of ROI selection: core-system baseline (BL), F&B, and FS.

### Forward and Backward Selection

**Figure 9:**
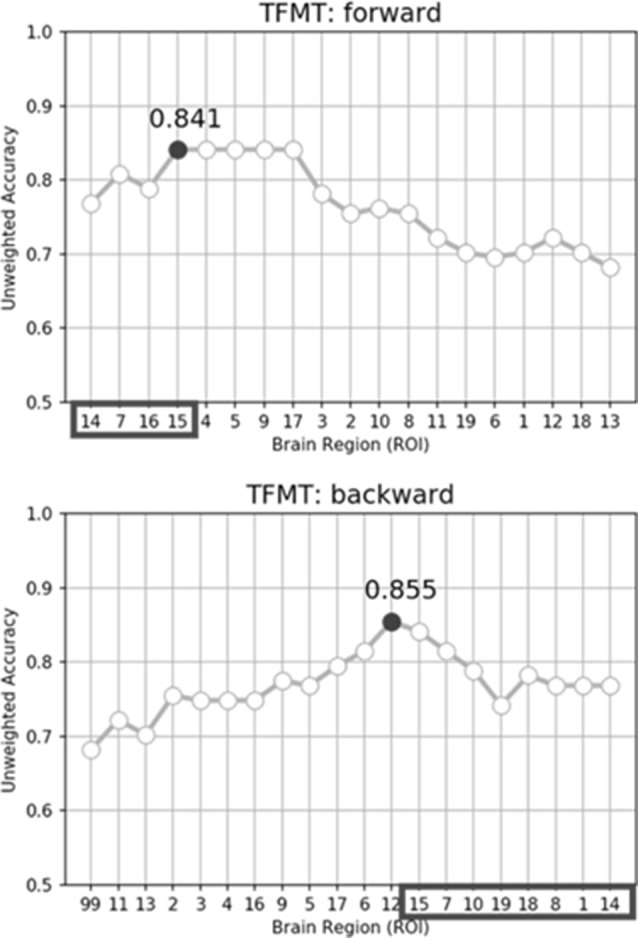
The unweighted classification accuracy on TFMT as a function of forward selection (top) and backward selection (bottom) of ROIs (see text for details). Black-outlined boxes highlighted the selected region-of-interest (ROI) that were significant.

While performing the forward selection, it is reasonable to take into consideration the attributes selected *via* the backward selection (Zhang, 2009). When achieved highest recognition accuracy in forward selection, the selected attributes were considered as influential ROIs. As illustrated in the top panel of Figure 9, with forward selection, the unweighted accuracy in predicting TFMT performance was 84.1% when ROI #14, #7, #16, and #15 were consecutively selected. Analogously, the backward selection of ROI #15, #7, #10, #19, #18, #8, #1, and #14 yielded 85.5% of accuracy after iteratively removing least useful brain regions. Across the two selection methods, the more robust ROIs were Calcarine(R; #7), OFA(L; #14), and OFA(R; #15; see Table 3), suggesting that the F&B scheme was more likely to pick out regions that are mostly involved in basic aspects of face processing.

### Feature Selection

**Figure 10:**
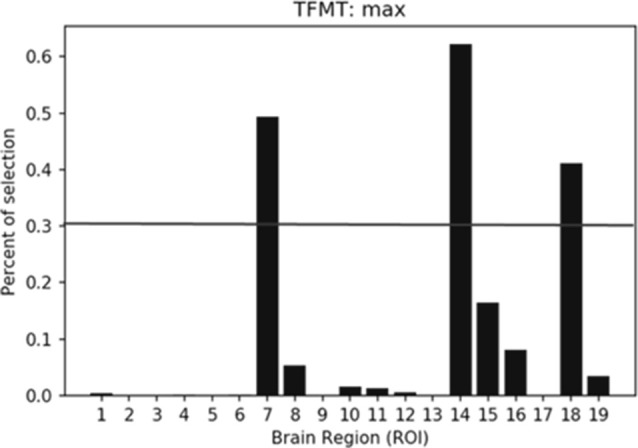
The percentiles of ROI by feature selection based on calculation of Fisher scores. The horizontal line represents the threshold (0.3) for final ROI selection (see text for details).

The percentiles of selected ROIs using ANOVA FS with TFMT are presented in Figure 10. We showed the selection percentile of 19 ROIs after recognizing target with FS, where the *x*-axis denotes the ROIs and the *y*-axis denotes the percentile for the selected region. The purpose of FS was to extract more effective ROIs, which could yield better performance with fewer numbers of features from the ROIs. Therefore, after taking into account BOLD activations of all 19 ROIs, we checked the result and set a percentile threshold of 0.3, and found that features from ROI #7, #14, and #18, namely, Calcarine(R), OFA(L), and pSTS(R), contributed more to the TFMT performance (see Figure 10).

### SVM Classification Accuracy

The prediction accuracy based on SVM classification between the three sets of ROI and feature extraction is shown in Table 4, which indicates that the average performance was best based on the combined result of F&B (74.2%), followed by FS (74.2%), and worst by the core-system baseline (BL; 65.6%). Taken together, these results suggest that basic and structural aspects of face processing can make substantial contribution to the performance on TFMT.

**Table 4:** The prediction accuracy of SVM classification on TFMT performance as a result of three sets of ROI compositions (face recognition ability by applying SVM, where BL, core-system baseline; F&B, forward and backward selection; FS, feature selection).

	TFMT			
Feature extraction	ROIs	Hit	CR	UA (%)
BL	ATL(L), ATL(R), FFA(L), FFA(R), OFA(L), OFA(R), pSTS(L), pSTS(R)	68.0	63.2	65.6
F&B	Cal(R), OFA(L), OFA(R)	80.0	68.4	74.2
FS	Cal(R), OFA(L), pSTS(L)	88.0	57.8	72.9

*Note: ROIs, regions of interest; CR, correct rejection; UA (%), percent unweighted average; ATL, anterior temporal lobe; FFA, fusiform face area; OFA, occipital face area; pSTS, posterior superior temporal sulcus; Cal, calcarine; R, right hemisphere; L, left hemisphere*.

## General Discussion

For more than four decades ever since Yin (1969) made his pioneering observation on the unique properties associated with face processing (i.e., the inversion effect), researchers have devised a set of well-tested tasks, including the component task, configural task, and composite tasks, for examining and tapping into various aspects of face processing (Rossion, 2008, 2009, 2013). On the other hand, over the past two decades, those who are intrigued by the neural and brain mechanisms underlying face processing have also proposed and investigated a complex network involving numerous brain regions for extracting and reading a variety of information from the human face (Haxby et al., 2000; Haxby and Gobbini, 2011; Freiwald et al., 2016). However, there appears to be an empirical gap between the scrutiny using relatively sophisticated behavioral tasks and the brain imaging research. The present study attempted to fill that gap by examining the extent to which brain activities of face-selective regions can predict performance in face processing and face memory, especially when non-Caucasian EA faces were involved.

Using the ROI approach, we first identified a set of brain regions that are deemed highly face-selective, including OFA, FFA, pSTS/fSTS, and vATL, and then correlated their activities with participants’ behavioral performances on the three face-processing tasks and the face memory test (i.e., TFMT). We found evidence indicating limited predictability from brain activities to behavioral performances. We consider a number of reasons that may account for the findings.

First, we used the one-back WM task, which has been a common practice in the extant literature on brain imaging, to assess and identify the core-system ROIs for face processing. The one-back task essentially requires the participants to maintain a memory trace of the currently displayed face image for a short period of time so that it can be retrieved and compared with the subsequently displayed face image. This demand on working memory in the one-back task, while useful in localizing face-selective brain regions, may have inadvertently obscured the predictability of activities from the selected brain regions on the performance of face-processing task. The main difference was that, for each of the behavioral tasks, we adopted a version where a pair of faces were displayed on each trial and participants had to engage in the processing according to the task demand. The fact that the face stimuli of concern were always presented simultaneously precluded or at least minimized the involvement of working memory, which was actually accorded well with the original intent of task design for having a pure demand on *perceptual*, rather than memorial, processing of faces.

A second possible reason bears on the possibilities of cultural differences in face processing in that most of the face stimuli that have been used so far for developing face-processing tasks involve the use of Caucasian faces. Although it is true that non-Caucasian faces (and databases) have been devised and used broadly in the literature, it has yet to reach systematic conclusions for comparing and contrasting the similarities and differences in processing faces of different ethnic and cultural origins. In fact, inquiry into the brain mechanisms may actually present a worthy opportunity for looking into such similarities as well as differences between participants of different cultural backgrounds. For example, it may well be possible that the core and extended systems that have been amply demonstrated with Caucasian participants using Caucasian faces may actually operate differently for, say, EA participants when they were processing EA faces, or an entirely different network may be involved in processing and extracting various information from a face. The results of our MVPA attempt appear to provide some support to the latter possibility in that the brain regions that were more viable in predicting performance in memorizing EA faces were those that appear to be involved in more basic aspects of structural processing of a face, rather than the more invariant aspects of face processing that have been demonstrated and argued for using Caucasian faces (Collins and Olson, 2014; Collins et al., 2016).

An intriguing, though somewhat remote, possibility that culture can influence face processing may involve the specific language, in particular the written language, developed and cultivated by people with shared cultural experiences. There has been ample evidence suggesting that face processing and word recognition may employ the same brain region of fusiform gyrus even though the functional organization for them are differentially weighted across the two hemispheres. More specifically, the fusiform area in the right hemisphere is relatively tuned for processing faces, whereas that in the left hemisphere is more calibrated for word recognition (for a critical review see Behrmann and Plaut, 2020).

The re-purposed use of evolutionally older brain regions (e.g., fusiform gyrus for face processing) to meet the requirements that arose from the ontogenesis of relatively new or novel cognitive functions (e.g., fusiform gyrus for word recognition) has been argued by a number of researchers from a variety of perspectives, such as neural recycling (Dehaene and Cohen, 2007), neural reuse (Anderson, 2010), language as shaped by the brain (Christiansen and Chater, 2016), and neuroconstructivism (Karmiloff-Smith, 2009). Most recently, Hernandez et al. (2019) have proposed a framework, called neurocomputational emergentism (or neuroemgentism for short) as a way to synthesize the existing approaches where combination of smaller elements can lead to a greater whole *via* nonlinear dynamic trajectories of development. While neuroemergentism is interesting as an explanatory framework in its present form, further research will be needed to attest its potential as a predictive framework (Marian and Hayakawa, 2019). We consider future research in the effect of cultural difference on face processing would match this expectation. For instance, how people cognitively nurtured in a predominantly logograph-based language (e.g., traditional Chinese) may employ brain mechanisms differently to process and remember faces than those primarily nurtured in an alphabet-based language should provide help validating the essential conjectures of neuroemergentism.

Finally, the possibility of hometown population density and gender differences can yet be another avenue whereby culture may exert is influence on face processing and memory. A recent study by Sunday et al. (2019) showed that hometown population density failed to predict participants’ performances on measures of face recognition ability regardless whether they included a learning component. Even so, they did find a pattern of gender differences modulated by hometown population density. The latter finding suggests that if the experience with faces in one’s hometown environment affect face recognition ability, the quality of such experiences rather than its sheer quantity would be crucial because men and women are likely to have different experiences with faces even when they grew up in the same environment. This line of reasoning can be extended to investigating how people with different cultural norm and background may process faces in disparate manners as those of different gender do. In conclusion, these and other possible reasons for explaining the discrepancy between findings from the extant literature and the present study call for future inquiry along the possibility of cultural influences on face processing and the associated brain mechanisms.

## Data Availability Statement

The datasets generated for this study are available on request to the corresponding author.

## Ethics Statement

The studies involving human participants were reviewed and approved by National Cheng Kung University Governance Framework for Human Research Ethics. The patients/participants provided their written informed consent to participate in this study.

## Author Contributions

GS and S-TH conceptualized and designed the behavioral and neuroimaging experiments. PC designed the computer programs for data collection and with BC analyzed brain imaging data. C-CL, W-TH, and FT performed the MVPA machine learning analyses.

## Conflict of Interest

The authors declare that the research was conducted in the absence of any commercial or financial relationships that could be construed as a potential conflict of interest.

## Footnotes

^1^http://marsbar.sourceforge.net/

## References

[B1] Anderson M. L. (2010). Neural reuse: a fundamental organizational principle of the brain. Behav. Brain Sci. 33, 245–266. 10.1017/s0140525x10000853 20964882

[B2] Andrews T. J. Schluppeck D. (2004). Neural responses to Mooney images reveal a modular representation of faces in human visual cortex. NeuroImage 21, 91–98. 10.1016/j.neuroimage.2003.08.023 14741646

[B3] Behrmann M. Plaut D. C. (2020). Hemispheric organization for visual object recognition: a theoretical account and empirical evidence. Perception 49, 373–404. 10.1177/0301006619899049 31980013 PMC9944149

[B6] Benton A. L. Sivan A. B. Hamsher K. D. S. Varney N. R. Spreen O. (1983). Contribution to Neuropsychological Assessment. New York, NY: Oxford University Press.

[B7] Benton C. P. Jennings S. J. Chatting D. J. (2006). Viewpoint dependence in adaptation to facial identity. Vision Res. 46, 3313–3325. 10.1016/j.visres.2006.06.002 16844181

[B8] Blais C. Jack R. E. Scheepers C. Fiset D. Caldara R. (2008). Culture shapes how we look at faces. PLoS One 3:e3022. 10.1371/journal.pone.0003022 18714387 PMC2515341

[B301] Brett M. Johnsrude I. S. Owen A. M. (2002). The problem of functional localization in the human brain. Nat. Rev. Neurosci. 3, 243–249. 10.1038/nrn756 11994756

[B9] Bruce V. Young A. (1986). Understanding face recognition. Br. J. Psychol. 77, 305–327. 10.1111/j.2044-8295.1986.tb02199.x 3756376

[B10] Bruce V. Young A. (2012). Face Perception. London, UK: Psychology Press.

[B12] Chen B. Y.-C. Cheng P. K.-H. Hung V. C.-S. Shyi G. C.-W. (2017). “Shared and non-shared neural mechanisms in processing dynamic transformation of expression and pose in faces: an fMRI study,” in Paper Presented at the 11th International Conference on Cognitive Science (Taipei, Taiwan).

[B13] Cheng P. K.-H. Hung V. Lin E. F.-Y. Shyi G. C.-W. Huang S.-T. T. (2016). Exploring brain mechanisms underlying individual differences in the effect of acquired familiarity on face learning and generalization. J. Vis. 16:908 10.1167/16.12.908

[B14] Cheng Y.-H. Shyi G. C.-W. Cheng P. K.-H. (2016). Age differences in face memory and face processing between younger and older adults in Taiwan. Chinese J. Psychol. 58, 233–262. 10.10.6129/CJP.20161008

[B15] Christiansen M. H. Chater N. (2016). Creating Language: Integrating Evolution, Acquisition and Processing. Cambridge, MA: MIT Press.

[B16] Collins J. A. Olson I. R. (2014). Beyond the FFA: the role of the ventral anterior temporal lobes in face processing. Neuropsychologia 61, 65–79. 10.1016/j.neuropsychologia.2014.06.005 24937188 PMC4122611

[B17] Collins J. A. Koski J. E. Olson I. R. (2016). More than meets the eye: The merging of perceptual and conceptual knowledge in the anterior temporal face area. Front. Hum. Neurosci. 10:189. 10.3389/fnhum.2016.00189 27199711 PMC4852584

[B18] Dash M. Liu H. (1997). Feature selection for classification. Intell. Data Anal. 1, 131–156. 10.3233/IDA-1997-1302

[B19] DeGutis J. Wilmer J. Mercado R. J. Cohan S. (2013). Using regression to measure holistic face processing reveals a strong link with face recognition ability. Cognition 126, 87–100. 10.1016/j.cognition.2012.09.004 23084178

[B20] Dehaene S. Cohen L. (2007). Cultural recycling of cortical maps. Neuron 56, 384–398. 10.1016/j.neuron.2007.10.004 17964253

[B21] Duchaine B. C. Nakayama K. (2006). The cambridge face memory test: results for neurologically intact individuals and an investigation of its validity using inverted face stimuli and prosopagnosic participants. Neuropsychologia 44, 576–585. 10.1016/j.neuropsychologia.2005.07.001 16169565

[B303] Duchaine B. Yovel G. (2015). A revised neural framework for face processing. Annu. Rev. Vis. Sci. 1, 393–416. 10.1146/annurev-vision-082114-035518 28532371

[B22] Eger E. Schyns P. G. Kleinschmidt A. (2004). Scale invariant adaptation in fusiform face-responsive regions. NeuroImage 22, 232–242. 10.1016/j.neuroimage.2003.12.028 15110013

[B23] Ewbank M. P. Andrews T. J. (2008). Differential sensitivity for viewpoint between familiar and unfamiliar faces in human visual cortex. NeuroImage 40, 1857–1870. 10.1016/j.neuroimage.2008.01.049 18343161

[B24] Farah M. J. Wilson K. D. Drain M. Tanaka J. N. (1998). What is “special” about face perception? Psychol. Rev. 105, 482–498. 10.1037/0033-295x.105.3.482 9697428

[B25] Fox C. J. Moon S.-Y. Iaria G. Barton J. J. S. (2009). The correlates of subjective perception of identity and expression in the face network: an fMRI adaptation study. NeuroImage 44, 569–580. 10.1016/j.neuroimage.2008.09.011 18852053 PMC2648406

[B26] Fox C. J. Iaria G. Barton J. J. S. (2009). Defining the face processing network: optimization of the functional localizer in fMRI. Hum. Brain Mapp. 30, 1637–1651. 10.1002/hbm.20630 18661501 PMC6870735

[B304] Freiwald W. Duchaine B. Yovel G. (2016). Face processing systems: From neurons to real-world social perception. Annu. Rev. Neurosci. 39, 325–346. 10.1146/annurev-neuro-070815-013934 27442071 PMC5345271

[B28] Gauthier I. Bukach C. M. (2007). Should we reject the expertise hypothesis? Cognition 103, 322–330. 10.1016/j.cognition.2006.05.003 16780825

[B29] Gauthier I. Tarr M. J. (1997). Becoming a “greeble” expert: exploring mechanism for face recognition. Vision Res. 37, 1673–1682. 10.1016/j.cognition.2006.05.003 9231232

[B30] Gobbini M. I. Haxby J. V. (2007). Neural systems for recognition of familiar faces. Neuropsychologia 45, 32–41. 10.1016/j.neuropsychologia.2006.04.015 16797608

[B31] Grill-Spector K. Kushnir T. Edelman S. Avidan G. Itzchak Y. Malach R. (1999). Differential processing of objects under various viewing conditions in the human lateral occipital complex. Neuron 24, 187–203. 10.1016/s0896-6273(00)80832-6 10677037

[B306] Haxby J. V. Gobbini M. (2007). The perception of emotion and social cues in faces. Neuropsychologia 45:1. 10.1016/j.neuropsychologia.2006.11.001 17109900

[B32] Haxby J. V. Gobbini M. I. (2011). “Distributed neural systems for face perception,” in Handbook of Face Perception, eds Calder A. Rhodes G. Johnson M. H. Haxby J. (Oxford: Oxford University Press), 93–110.

[B307] Haxby J. V. Hoffman E. A. Gobbini M. I. (2000). The distributed human neural system for face perception. Trends Cogn. Sci. 4, 223–233. 10.1016/s1364-6613(00)01482-0 10827445

[B305] Haxby J. V. Connolly A. C. Guntupalli J. S. (2014). Decoding neural representational spaces using multivariate pattern analysis. Annu. Rev. Neurosci. 37, 435–456. 10.1146/annurev-neuro-062012-170325 25002277

[B33] Henrich J. Heine S. J. Norenzayan A. (2010). The weirdest people in the world? Behav. Brain Sci. 33, 61–135. 10.1017/S0140525X0999152X 20550733

[B34] Hernandez A. E. Claussenius-Kalman H. L. Ronderos J. Castilla-Earls A. P. Sun L. Weiss S. D.. (2019). Neuroemergentism: a framework for studying cognition and the brain. J. Neurolinguistics 49, 214–223. 10.1016/j.jneuroling.2017.12.010 30636843 PMC6326375

[B35] Hole G. J. (1994). Configurational factors in the perception of unfamiliar faces. Perception 23, 65–74. 10.1068/p230065 7936977

[B36] Hole G. Bourne V. (2010). Face Processing: Psychological, Neuropsychological, and Applied Perspectives. New York, NY: Oxford University Press.

[B37] Huang S.-T. T. Lee M. C. Lee L. W. Chan Y. T. Tsai H. T. (2014). Taiwan corpora of Chinese emotional stimuli database: the study of emotional prosody. Chinese J. Psychol. 56, 437–452. 10.6129/CJP.20140814

[B38] Kanwisher N. Barton J. J. S. (2011). “The functional architecture of the face system: integrating evidence from fMRI and patient studies,” in Handbook of Face Perception, eds Calder A. Rhodes G. Johnson M. H. Haxby J. (Oxford: Oxford University Press), 111–129.

[B39] Kanwisher N. McDermott J. Chun M. M. (1997). The fusiform face area: a module in human extrastriate cortex specialized for face perception. J. Neurosci. 17, 4302–4311. 10.1523/JNEUROSCI.17-11-04302.1997 9151747 PMC6573547

[B40] Karmiloff-Smith A. (2009). Nativism versus neuroconstructivism: rethinking the study of developmental disorders. Dev. Psychol. 45, 56–63. 10.1037/a0014506 19209990

[B41] Kelly D. J. Liu S. Rodger H. Miellet S. Ge L. Caldara R. (2011). Developing cultural differences in face processing. Dev. Sci. 14, 1176–1184. 10.1111/j.1467-7687.2011.01067.x 21884332

[B42] Kelly D. J. Miellet S. Caldara R. (2010). Culture shapes eye movements for visually homogenous object. Front. Psychol. 1:6. 10.3389/fpsyg.2010.00006 21833189 PMC3153738

[B43] Kimchi R. (1992). Primacy of wholistic processing and global/local paradigm: a critical review. Psychol. Bull. 112, 24–38. 10.1037/0033-2909.112.1.24 1529037

[B44] Liu J. Harris A. Kanwisher N. (2002). Stages of processing in face perception: an MEG study. Nat. Neurosci. 22, 203–211. 10.1038/nn909 12195430

[B45] Liu J. Harris A. Kanwisher N. (2010). Perception of face parts and face configurations: an fMRI study. J. Cogn. Neurosci. 22, 203–211. 10.1162/jocn.2009.21203 19302006 PMC2888696

[B46] Marian V. Hayakawa S. (2019). Neuroemergentism: at the intersection of ontogeny and phylogeny. J. Neurolinguistics 49, 252–254. 10.1016/j.jneuroling.2018.05.001 30828132 PMC6391879

[B47] Maurer D. Le Grand R. Mondloch C. J. (2002). The many faces of configural processing. Trends Cogn. Sci. 6, 255–260. 10.1016/s1364-6613(02)01903-4 12039607

[B49] McKone E. (2010). “Face and object recognition: how do they differ?,” in Tutorials in Visual Cognition, ed. Coltheart V. (New York, NY: Psychology Press), 261–303.

[B50] McKone E. Yovel G. (2009). Why does picture-plane inversion sometimes dissociate perception of features and spacing in faces and sometimes not? Toward a new theory of holistic processing. Psychon. Bull. Rev. 16, 778–797. 10.3758/pbr.16.5.778 19815781

[B51] Mondloch C. J. Le Grand R. Maurer D. (2002). Configural face processing develops more slowly than featural face processing. Perception 31, 553–566. 10.1068/p3339 12044096

[B53] Moret-Tatay C. Baixauli-Fortea I. Sevilla M. Irigaray T. Q. (2020). Can you identify these celebrities? A network analysis on differences between word and face recognition. Mathematics 8:699 10.3390/math8050699

[B54] Norman K. A. Polyn S. M. Detre G. J. Haxby J. V. (2006). Beyond mind-reading: multi-voxel pattern analysis of fMRI data. Trends Cogn. Sci. 10, 424–430. 10.1016/j.tics.2006.07.005 16899397

[B57] Richler J. J. Cheung O. S. Gauthier I. (2011). Holistic processing predicts face recognition. Psychol. Sci. 22, 464–471. 10.1177/0956797611401753 21393576 PMC3077885

[B55] Richler J. J. Gauthier I. (2013). When intuition fails to align with data: a reply to (Rossion (2013)). Vis. Cogn. 21, 254–276. 10.1080/13506285.2013.796035 24307858 PMC3845673

[B56] Richler J. J. Gauthier I. (2014). A meta-analysis and review of holistic face processing. Psychol. Bull. 140, 1281–1302. 10.1037/a0037004 24956123 PMC4152424

[B58] Ross D. A. Richler J. J. Gauthier I. (2014). Reliability of composite task measurements of holistic processing. Behav. Res. Methods 47, 736–743. 10.3758/s13428-014-0497-4 24961957 PMC4276735

[B59] Rossion B. (2008). Picture-plane inversion leads to qualitative changes of face perception. Acta Psychol. 128, 274–289. 10.1016/j.actpsy.2008.02.003 18396260

[B60] Rossion B. (2009). Distinguishing the cause and consequence of face inversion: the perceptual field hypothesis. Acta Psychol. 132, 300–312. 10.1016/j.actpsy.2008.02.003 19747674

[B61] Rossion B. (2013). The composite face illusion: a whole window into our understanding of holistic face perception. Visual Cogn. 21, 139–253. 10.1080/13506285.2013.772929

[B64] Shyi G. C.-W. He H.-M. (2011). Effects of familiarity and expression variation on face recognition and generalization. Chinese J. Psychol. 53, 437–470. 10.6129/CJP.2011.5304.08

[B65] Shyi G. C.-W. Huang T. S.-T. Yeh C.-Y. (2013). Taiwan corpora of Chinese emotions and relevant psychophysiological data: a college-student database of facial expression for basic emotions. Chinese J. Psychol. 55, 455–474. 10.6129/CJP.20121226

[B66] Sunday M. A. Patel P. A. Dodd M. D. Gauthier I. (2019). Gender and hometown population density interact to predict face recognition ability. Vision Res. 163, 14–23. 10.1016/j.visres.2019.08.006 31472340

[B67] Tanaka J. W. Farah M. J. (1993). Parts and wholes in face recognition. Q. J. Exp Psychol. 46, 225–245. 10.1080/14640749308401045 8316637

[B68] Tanaka J. W. Farah M. J. (2003). “The holistic representation of faces,” in Perception of Faces, Objects and Scenes: Analytic and Holistic Processes, eds Peterson M. A. Rhodes G. (New York, NY: Oxford University Press), 53–74.

[B69] Tanaka J. W. Gordon I. (2011). “Features, configuration and holistic face processing,” in The Oxford Handbook of Face Perception, eds Calder A. Rhodes G. Johnson M. H. Haxby J. (Oxford, UK: Oxford University Press), 177–194.

[B308] Tzourio-Mazoyer N. Landeau B. Papathanassiou D. Crivello F. Etard O. Delcroix N.. (2002). Automated anatomical labeling of activations in SPM using a macroscopic anatomical parcellation of the MNI MRI single-subject brain. NeuroImage 15, 273–289. 10.1006/nimg.2001.0978 11771995

[B70] Warrington E. K. (1984). Recognition Memory Test. Windsor, UK: NFER-Nelson.

[B71] Winston J. S. Henson R. N. A. Fine-Goulden M. R. Dolan R. J. (2004). fMRI-adaptation reveals dissociable neural representations of identity and expression in face perception. J. Neurophysiol. 92, 1830–1839. 10.1152/jn.00155.2004 15115795

[B72] Yang J. Shyi G. C.-W. (2010). Face recognition and its developmental differences: a multi-level review of literature. Res. Appl. Psychol. 46, 153–230.

[B309] Yarbus A. L. (1965). Role of eye movements in the visual process. Nauka.

[B310] Yin R. K. (1969). Looking at upside-down faces. J. Exp. Psychol. 81, 141–145. 10.1037/h0027474

[B74] Young A. W. Hellawell D. Hay D. C. (1987). Configurational information in face perception. Perception 16, 747–759. 10.1068/p160747 3454432

[B75] Yovel G. Kanwisher N. (2005). The neural basis of the behavioral face-inversion effect. Curr. Biol. 15, 2256–2262. 10.1016/j.cub.2005.10.072 16360687

[B76] Zhang T. (2009). “Adaptive forward-backward greedy algorithm for sparse learning with linear models,” in Advances in Neural Information Processing Systems, eds Koller D. Schuurmans D. Bengio Y. Bottou L. (Cambridge, MA: MIT Press), 1921–1928.

